# The genomes of the yaws bacterium, *Treponema pallidum* subsp. *pertenue*, of nonhuman primate and human origin are not genomically distinct

**DOI:** 10.1371/journal.pntd.0011602

**Published:** 2023-09-13

**Authors:** Klára Janečková, Christian Roos, Pavla Fedrová, Nikola Tom, Darina Čejková, Simone Lueert, Julius D. Keyyu, Idrissa S. Chuma, Sascha Knauf, David Šmajs

**Affiliations:** 1 Department of Biology, Faculty of Medicine, Masaryk University, Brno, Czech Republic; 2 Deutsches Primatenzentrum GmbH, Leibniz-Institute for Primate Research, Göttingen, Germany; 3 Department of Biomedical Engineering, Brno University of Technology, Brno, Czech Republic; 4 Institute of International Animal Health/One Health, Friedrich-Loeffler-Institut, Federal Research Institute for Animal Health, Greifswald—Insel Riems, Germany; 5 Tanzania Wildlife Research Institute (TAWIRI), Arusha, Tanzania; 6 Department of Veterinary Medicine and Public Health, College of Veterinary and Medical Sciences, Sokoine University of Agriculture, Morogoro, Tanzania; University of Oxford, THAILAND

## Abstract

**Background:**

*Treponema pallidum* subsp. *pertenue* (*TPE*) is the causative agent of human yaws. Yaws is currently reported in 13 endemic countries in Africa, southern Asia, and the Pacific region. During the mid-20th century, a first yaws eradication effort resulted in a global 95% drop in yaws prevalence. The lack of continued surveillance has led to the resurgence of yaws. The disease was believed to have no animal reservoirs, which supported the development of a currently ongoing second yaws eradication campaign. Concomitantly, genetic evidence started to show that *TPE* strains naturally infect nonhuman primates (NHPs) in sub-Saharan Africa. In our current study we tested hypothesis that NHP- and human-infecting *TPE* strains differ in the previously unknown parts of the genomes.

**Methodology/Principal findings:**

In this study, we determined complete (finished) genomes of ten *TPE* isolates that originated from NHPs and compared them to *TPE* whole-genome sequences from human yaws patients. We performed an in-depth analysis of *TPE* genomes to determine if any consistent genomic differences are present between *TPE* genomes of human and NHP origin. We were able to resolve previously undetermined *TPE* chromosomal regions (sequencing gaps) that prevented us from making a conclusion regarding the sequence identity of *TPE* genomes from NHPs and humans. The comparison among finished genome sequences revealed no consistent differences between human and NHP *TPE* genomes.

**Conclusion/Significance:**

Our data show that NHPs are infected with strains that are not only similar to the strains infecting humans but are genomically indistinguishable from them. Although interspecies transmission in NHPs is a rare event and evidence for current spillover events is missing, the existence of the yaws bacterium in NHPs is demonstrated. While the low risk of spillover supports the current yaws treatment campaign, it is of importance to continue yaws surveillance in areas where NHPs are naturally infected with *TPE* even if yaws is successfully eliminated in humans.

## Author summary

In this study, we focused on yaws, a neglected tropical disease, and its causative agent, bacterium *Treponema pallidum* subsp. *pertenue* (*TPE*). Yaws is currently reported in 13 endemic countries in Africa, southern Asia, and the Pacific region. It predominantly infects children and causes papilloma, ulcers and, if untreated, progresses into disfiguring deformation of the bone and cartilage. The second yaws eradication campaign, known as the Morges strategy, is currently ongoing and led by World Health Organization (WHO). There is, however, accumulating genetic evidence that *TPE* strains naturally infect nonhuman primates (NHPs) in Sub-Saharan Africa. Here, we wanted to find out if the most serious argument–nonhuman and human infecting strains of *TPE* differ in the previously unknown parts of their genomes, that was raised by the scientific community against nonhuman primates as a source for human yaws infection–is true. Our findings highlight NHPs as a possible source for human yaws, although in this study, we cannot provide insight into the functional aspects of spillover between humans and NHPs. Moreover, these findings underline that *Treponema pallidum* subsp. *pertenue* can only be eliminated in humans, while the bacterium is still present in nonhuman primates in Sub-Saharan Africa. Whether or not human and nonhuman primate infecting strains are epidemiologically linked is subject to ongoing research. Nevertheless, our results highlight the need for a long-term yaws surveillance program in areas where the infected NHP populations occur.

## Introduction

*Treponema pallidum* is the causative agent of syphilis (subsp. *pallidum*, *TPA*), bejel (subsp. *endemicum*, *TEN*), and human yaws (subsp. *pertenue TPE*) [1,2]. Compared to syphilis with its worldwide incidence, human yaws currently infects mainly children in 13 endemic countries in Africa, southern Asia, and the Pacific region [3]. The endemic character of yaws infection, the significant drop in prevalence after the first eradication campaign during the mid-20^th^ century [3], the believed absence of a nonhuman reservoir, and new treatment options with a single oral dose of azithromycin [4–6] triggered the currently ongoing second yaws eradication campaign known as the Morges strategy [7].

Concomitantly with the introduction of the Morges strategy, genetic evidence started to show that *TPE* strains naturally infect some African nonhuman primates (NHPs) [8–14]. The work of Chuma *et al*. [15] provided genetic evidence of *TPE* infection in almost a hundred samples from six nonhuman primates species collected in Tanzania, Ethiopia, and the Republic of the Congo.

Despite increasing evidence of NHP *TPE* infection, one of the most important questions for yaws eradication is whether NHP infecting strains are genomically distinct to human infecting strains and if NHPs can serve as a potential source of human infection. The question remains unanswered, partly because we were short on complete genome sequences of *TPE* of NHP origin. Presently, there are only two complete NHP *TPE* genomes (strain Fribourg-Blanc [10] and strain LMNP-1 [11]); additionally, there are 14 draft genomes of varying quality [11,12,16]. In this study, we aimed to close this knowledge gap and therefore sequenced ten more genomes of NHP origin and finalized eight of them, lifting the total number of available complete genomes of NHP *TPE* to a total of ten, and compared all ten NHP *TPE* genomes to the eight completed *TPE* genomes of human origin. We hypothesized that the NHP strains were not genetically distinct from the human strains.

## Materials and methods

### Sample selection

**Fig 1:**
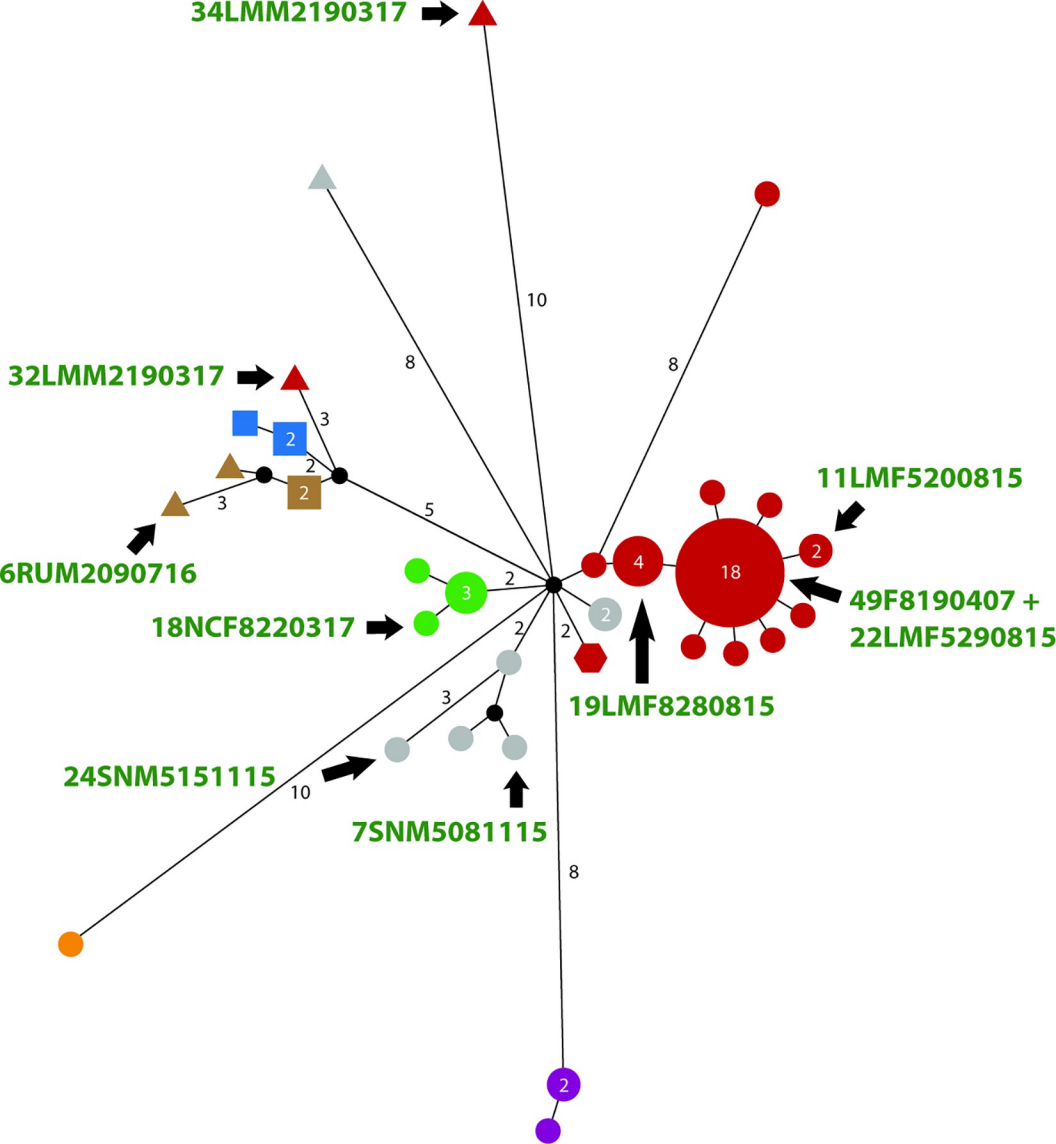
*TPE* strains of NHP origin from Tanzania chosen for whole-genome sequencing. The figure is taken and modified from Chuma *et al*. [15]. Original figure (https://www.nature.com/articles/s41598-019-50779-9/figures/1) was licensed under a Creative Commons Attribution 4.0 International License (https://creativecommons.org/licenses/by/4.0/). Median-joining network using 1,773 bp–long concatemer of TP0488 and TP0548 loci from 57 Tanzanian NHPs samples. The number of nucleotide differences, when >1, are shown close to branches. Inferred allelic variants (median vectors) are shown as small black circles. If contiguous, indels were considered as single events only. The number of individual sequence variants, when > 1, are shown inside the circles and reflected by circle size. Color-code based on origin: blue–Issa Valley (n = 3), orange–Tarangire National Park (NP) (n = 1), brown–Ruaha NP (n = 4), red–Lake Manyara NP (n = 34), grey–Serengeti NP (n = 7), green–Ngorongoro Conservation Area (n = 5), violet–Gombe NP (n = 3). Geometric form according to the species: circle–*Papio anubis* (n = 46), square–*Papio cynocephalus* (n = 5), triangle–*Chlorocebus pygerythrus* (n = 5), hexagon–*Cercopithecus mitis* (n = 1). Sequenced samples are shown by black arrows. One of the arrows labels two strains (49F8190407 and 22LMF5290815).

No live animals were sampled for this study. All NHP samples were selected from our previous studies [9,17], where the interested reader will also find the ethical statements and further details regarding DNA extraction methods. Samples collected included whole blood, genital swabs and skin tissue biopsies. Further information about the samples can be found in Results section. The selection of suitable samples from our collection followed a process of quality checks and selection for diversity based on (1) genetic diversity based on TP0488 and TP0548 sequencing, (2) the sampled NHP species, (3) the geographic origin of the sample, and (4) the available quality and amount of *TPE* DNA. Further details are shown in Fig 1 and described below in the downstream processing of DNA samples.

All NHP *TPE* isolates analyzed in this study are shown in Table 1. Seven samples originated from olive baboons (*Papio anubis*), and three samples were collected from vervet monkeys (*Chlorocebus pygerythrus*). Of these, we were able to generate complete genome sequences from five *P*. *anubis* and three *C*. *pygerythrus* infecting strains.

**Table 1:** Genome regions in the assembled genomes that were checked and filled using Sanger sequencing.

Strain	Number of gaps	Total length of gaps (nt)	Genome length (nt)	Percentage of genome finished by Sanger sequencing (%)
6RUM2090716	8	5,073	1,140,284	0.44
7SNM5081115	9	6,156	1,139,594	0.54
18NCF8220317	14	11,387	1,139,654	1.00
19LMF8280815	27	11,851	1,139,879	1.04
22LMF5290815	12	6,426	1,140,667	0.56
24SNM5151115	14	10,542	1,140,433	0.92
32LMM2190317	14	9,864	1,140,113	0.87
34LMM2190317	20	9,898	1,140,506	0.87

### Downstream processing of DNA samples

Based on our previous work, we used confirmed *TPE* positive DNA samples with moderate to high polymerase I (*polA*) gene copy numbers [9,17]. Subsequently, we checked DNA integrity by running a long-range PCR that targeted a 4,835 bp long region of the *tpr*C gene using primers (ES-42F and TPI-11A-R) and previously published PCR conditions [18]. The subset of samples was then evaluated for their total DNA yield and diversity based on our multi-locus sequence typing study [15].

We selected samples representing different haplotypes and subsequently enriched the bacterial DNA using Looxter Enrichment Kits (Analytik Jena, Jena, Germany) following the manufacturer’s protocol, or the bacterial DNA was enriched using *Dpn*I restriction enzyme [18]. After DNA-target enrichment, qPCR was performed to target the housekeeping gene *c-myc*, using the protocol described for baboon samples [9]. We used *c-myc* as a marker of the genomic (host) DNA content of the sample and used only those samples with a *polA*:*c-myc* ratio > 1:1,000 for whole-genome sequencing. Eventually, we were able to include samples from two different NHP species, *Chlorocebus pygerythrus* (vervet monkey) and *Papio anubis* (olive baboon), which had been collected in protected areas in Tanzania, i.e., Lake Manyara National Park (NP), Ngorongoro Conservation Area, Serengeti NP, and Ruaha NP. Further details on the samples are in Table 1.

At this point, we used different methods to generate the genomes. While five samples (6RUM2090716, 7SNM5081115, 22LMF5290815, 24SNM5151115, 49F8190407) had the target DNA enriched using hybridization capture for next-generation sequencing (NGS, myBaits, Arbor Biosciences, USA) (SK lab), the other five samples (18NCF8220317, 19LMF8280815, 32LMM2190317, 34LMM2190317, 11LMF5200815) were subject to whole genome amplification (WGA, DS lab) using REPLI-g Single Cell Kits (QIAGEN, Hilden, Germany) or REPLI-g Mini Kits (QIAGEN) using the manufacturer’s protocol. Amplified samples were purified using SPRISelect beads (Beckman Coulter, Indianapolis, USA).

### Library preparation and in-solution capture

Samples that were not whole genome amplified were *TPE* DNA enriched using hybridization capture for targeted NGS (myBaits, Arbor Biosciences, Ann Arbor, USA). In general, we followed the method described in our previous publication [11]. In brief, we used the NEBNext Ultra II FS DNA Library Prep Kit for Illumina and followed the manufacturer’s guidance. We followed the protocol for inputs ≥ 100–500 ng DNA and targeted a fragment size of 300–700 bps during the enzymatic digest. Subsequently, fragments were adapter-ligated and size selected using AMPure XP beads according to the NEB protocol. Where appropriate, DNA concentration was measured using a Qubit dsDNA High Sensitivity kit (ThermoFisher Scientific, Waltham, USA). The library was finalized through single index PCR enrichment of 15 μl adaptor-ligated DNA fragments using the Universal PCR primer (i5 Primer) in combination with the respective Index Primer (i7 Primer). The cycling conditions followed the recommendations provided by NEB. Next, PCR reactions were cleaned using AMPure XP beads following the standard protocol. Afterward, libraries were quality checked using a Bioanalyzer 2000 (Agilent, Palo Alto, USA) and quantified using the NEBNext Library Quant Kit for Illumina.

The capture of *TPE* DNA followed the myBaits Hybridization Capture for Targeted NGS Manual 4.0 using custom-made RNA baits (Arbor Biosciences, Ann Arbor, USA). Baits were designed based on 16 submitted *TPE* genomes (GenBank; human: CP002374.1, NC_016842.1, CP024750.1, NC_016848.1, NC_016843.1, CP024088.1, CP024089.1, and eight NHP: NC_021179.1 plus the genomes published by Knauf *et al*. [11]). Briefly, 80-nt baits with a 2x tiling density were designed after converting N-bases, in runs of ten or less, to Ts and removal of alignment gaps. Simple repeats were soft masked using RepeatMasker [19] and baits were collapsed at the 96% identity level. All bait sequences were BLASTed against the genomes of the olive baboon (*Papio anubis*, UCSC Genome Accession ID: GCF_000264685.3) and the African green monkey (*Chlorocebus sabaeus*; UCSC Genome Accession ID: GCA_000409795.2) with no hits against either genome.

Following library denaturation and adapter-blocking, target library molecules were allowed to hybridize with their complementary bait. Incubation times were set to 16 h and 30 min. Subsequently, bait-target hybrids were bound to streptavidin-coated magnetic beads, which allowed us to separate the desired target DNA molecules from non-target DNA through three washing steps. This was followed by resuspension of the library in 30 μl of 10 mM Tris-Cl, 0.05% TWEEN-20 solution (pH 8.0–8.5), and library amplification using KAPA HiFi Hot Start polymerase. Here, we followed the protocol and used 14 amplification cycles. After magnetic removal of the beads, the supernatant was purified using the SPRI cleanup method with NEBNext Sample Purification Beads (x 0.7; New England Biolabs GmbH, Frankfurt am Main, Germany) according to NEB’s protocol. The SPRI cleanup product was 5-fold diluted in 0.1X TE buffer and quantified on a Qubit 3.0 (ThermoFisher Scientific, Waltham, USA). In addition, 1 μl of the undiluted SPRI cleanup product was quality checked on the Bioanalyzer. Library quantification was performed using the qPCR NEBNext Quant Kit for Illumina (New England Biolabs, Ipswitch, USA) following NEB’s recommended protocol. To increase the yield of target DNA, we reamplified the library from our first hybridization capture run using KAPA HiFi Hot Start polymerase in combination with Illumina adapter specific primers (sense 5’-AAT GAT ACG GCG ACC ACC GA-3’ and antisense 5’-CAA GCA GAA GAC GGC ATA CGA-3’). Cycling conditions were 2 min at 98°C followed by five cycles of 20 sec at 98°C, 30 sec at 60°C, and 45 sec at 72°C. The last step was a final phase of 5 min at 72°C. Next, the library product was quantified using Qubit 3.0 and again cleaned using the SPRI cleanup method as described previously. After quantification (Qubit 3.0) and a quality check on a Bioanalyzer, we initiated a second round of hybridization capturefollowing the above-described methods. The enriched 10 nM DNA libraries were pooled and sent out for sequencing.

### DNA sequencing

Samples were processed using different platforms. Sample 22LMF5290815 was sequenced on an Illumina’s MiSeq, samples 19LMF8280815, 32LMM2190317, 34LMM2190317, and 18NCF8220317 on an Illumina’s HiSeq 2500, and samples 24SNM5151115, 7SNM5081115, 6RUM2090716, and 49F8190407 were sequenced by both MiSeq and HiSeq 2500 platforms. Sample 11LMF5200815 was amplified using the pooled segment genome sequencing (PSGS) method [20–22] and subsequently sequenced on a MiSeq. The sequencing platforms generated 150-bp (HiSeq 2500) or 250-bp (MiSeq) paired-end reads. We used the sequencing service of the Transcriptome and Genome Analysis Laboratory at the University Medical School Göttingen, Germany.

### Bioinformatic analysis

The bioinformatic analysis of sequencing data was performed according to the workflow described previously [23] with several minor upgrades. The quality check of the raw reads was performed using FastQC (v0.11.5, [24]) and followed by pre-processed using Cutadapt (v2.6, [25]). The total set of pre-processed reads was mapped against the *TPE* strain LMNP-1 (GenBank CP021113.1) genome as a reference using the BWA MEM tool [26]/ BWA MEM2 (v2.2.1, [27]).

Post-processing of the mapping was performed with SAMtools (v1.9, [28]), Picard (v2.23.8, [29]), GATK (v3.7, [30]), and NGSUtils/bamutils (v0.5.9, [31]). Specifically, reads were realigned around indels, duplicated reads were removed, and low-quality mappings were filtered out using parameters for (1) mismatches (maximum of 5% of mismatches and maximum of 5 mismatches), (2) very short alignments (minimum of 35 bp mapping measured on the individual read), (3) soft-clipped bases (maximum of 5% of soft-clipped bases, no reads with hard-clipping), (4) mapping quality < 40 (MAPQ < 40), and (5) presence of singletons. These criteria were applied in order to get a high quality sequences and to eliminate the potential differences in the sequencing protocols used. MiSeq and HiSeq paired-reads were merged using SAMtools. The genome consensus sequence was determined based on at least three good-quality aligned reads using Picard tools.

To obtain improved broad coverage and more precise variant calling results, especially in the regions of paralogous genes and repeats, reference mapping was complemented by two *de-novo* assembly approaches, including (1) SPAdes [32] and (2) GFinisher [33]. As an input for *de-novo* assembly, unfiltered reads aligned to the reference genome were used. Contigs generated by 2 *de-novo* assembly approaches were mapped onto the consensus generated in the previous step (consensus based on short reads aligned to LMNP-1 genome by BWA) using minimap2 [34], and non-specific alignments (MAPQ < 10) were removed. Following this, contigs were mapped on consensus acquired by mapping of short reads. Consensus calling was then done to correct the consensus.

Variants that represented differences between the BWA-based consensus and the contigs from any of the *de-novo* assembly approaches were re-sequenced using Sanger sequencing. This also applied to regions covered by *de-novo* assembled contigs but not covered by reference mapping (coverage < 4). Furthermore, regions insufficiently covered by either short read mapping (coverage < 4) or *de-novo* assembly (coverage 0) were also re-sequenced.

For identification of the variants and insufficiently covered regions, SAMtools, bcftools [35], and BEDTools [36] were used.

For Sanger re-sequencing, specifically tailored amplicons were used. For quality control and evaluation of completeness of the assembly, each step of our described workflow was manually checked using IGV [37] and QUAST [38].

SRA data for genomes for extended phylogenetic tree were downloaded from NCBI. SRA Toolkit (v2.10.0, http://ncbi.github.io/sra-tools/) was used to extract reads from SRA files. The quality check of the raw reads was performed using FastQC (v0.11.5, [24]). To identify adapter sequences, BBMap was used (v38.42, [39]) and raw reads were preprocessed using Cutadapt (v2.6, [25]). Subsequently, reads were mapped to the reference genome (*TPE* strain LMNP-1 (GenBank CP021113.1)) and post-processing and filtering was performed as described above.

### Genome gap filling

In each sequenced genome, gaps were identified and subsequently filled using PCR amplification and Sanger sequencing (Table 1).

Sequence gaps were amplified using TaKaRa PrimeSTAR GXL DNA Polymerase (Clontech, Mountain View, USA) in 40 μl PCR reactions. Details can be found in S1 Data–List of gaps + primers, where we present all sequenced regions and primer sequences. The reaction mix was made up of 8 μl GXL buffer, 3.2 μl dNTP mix (2.5 μM each), 3 μl DNA template, 0.8 μl PrimeStar GXL polymerase, 1.5 μl of each primer (10 μM) and 22 μl H_2_O. PCR was done using a Veriti 96-Well Fast Thermal Cycler with the following protocol: 94°C for 1 min, 8 cycles at 98°C for 10 s, 68°C (with TouchDown, temperature lowered 1°C after each cycle) for 15 s and 68°C for 6 min, 35 cycles at 98°C for 10 s, 61°C for 15 s, and 68°C for 6 min. The final extension was at 68°C for 7 min. In regions where nested PCR was applied, both steps were run under the above-described conditions. PCR products were checked for the correct fragment size on a 1% agarose gel. Subsequently, PCR products of the correct size were purified using QIAquick PCR Purification kits (QIAGEN, Hilden, Germany) following the manufacturer’s instructions. DNA was quantified using a NanoDrop ND-1000 (NanoDrop, Wilmington, USA). In a final step, prior to sequencing, each amplicon sample was mixed with 5 μl primer (5 μM) of the respective sequencing primer, after which it was Sanger sequenced using the services of GATC Biotech AG (Konstanz, Germany). The quality checks and analysis of the obtained sequences, as well as the final construction of the respective *TPE* genome, was done using SeqMan NGen (DNASTAR, Madison, USA). Again, using the LMNP-1 (CP021113.1) strain of the *TPE* genome as a reference.

### Sequence determination of *arp* (TP0433), TP0470, and *rrn* operons

The number of repeats in the acid-repeat protein (*arp*) gene (TP0433) and in TP0470 gene was determined by separate PCR amplification using three sets of primers (Table 2) for *arp*, and Bab32-LinOutL 5’ -ACC GCT ACA AAG AGG ATA GG-3’ and Bab32-LinOutR 5’ -GCT CTT TCT TTG TGC GTA GA-3’ for TP0470. All reactions used 0.8 μl TaKaRa PrimeSTAR GXL DNA Polymerase, 8 μl 5x PS GXL buffer, 3.2 μl dNTP Mix, 1.5 μl of each primer (10μM), 22 μl RNase free H_2_O, and 3 μl DNA template. PCR was run using a Veriti 96-Well Fast Thermal Cycler with the following protocol: 94°C for 1 min, 8 cycles at 98°C for 10 s, 68°C (with TouchDown, temperature lowered 1°C after each cycle) for 15 s and 68°C for 1 min, 35 cycles at 98°C for 10s, 61°C for 15 s as well as 68°C for 1 min. PCR ended with a final extension step at 68°C for 7 min. The resulting DNA segments were checked for correct size on 1% agarose gel, and Sanger sequenced.

**Table 2:** Primers used for *arp* sequencing.

Primer name	Primer sequence	Samples
32Brep-F1	5‘-CGT TTG GTT TCC CCT TTG TC	22LMF5290815, 24SNM5151115, 6RUM2090716, 7SNM5081115
32Brep-R1	5‘-CAT ACG AAG CAG CCA TCC CAC	
Bab29-LinOutL	5‘-ACA CTG CTG TTG AAA TTA GC	19LMF8280815, 18NCF8220317
Bab29-LinOutR	5‘-GAG CAT TGT CCA CCG TTA CG	
TP_arp_S_Harper2008	5‘-AGC GTG ATC CTC TGT CAT CC	32LMM2190317, 34LMM2190317
TP_arp_AS_Harper2008	5‘-TTG GGA GCT GAG TTG GAA AC	

For the samples 22LMF5290815, 24SNM5151115, and 6RUM2090716, Sanger sequencing reads were not long enough to accurately determine the number of repeats in the TP0470 gene. We, therefore, sequenced those *arp* products using MinION Oxford Nanopore Technologies (Oxford, UK). The sequencing library was prepared using the 1D Native barcoding genomic DNA protocol with EXP-NBD103 and SQK-LSK108 kits (Oxford Nanopore Technologies) according to the manufactures’ instructions. The prepared library was loaded to the Nanopore MinION Spoton flow cell (FLO-MIN106D, version R9) and sequenced for 48 hours. Base-calling and barcoding were done by Guppy (v4.4.1, Oxford Nanopore Technologies), using the high accuracy base-calling mode and q-score 7. Minimap2 (v2.17, [34]) was used to map reads to references; map reads of a known size (4–6kbp) were selected by SAMtools (v0.1.20, [28]) and Picard (v 2.8.1, [29]). The 50 longest reads were evaluated in the EditSeq program (Lasergene DNASTAR, Madison, USA), and the most frequent variant was used to determine the number of repeats. The position of *rrn* spacer patterns in all samples was determined using the previously published approach [21].

### Data analysis

Statistical analyses of the numbers of repeats in genes TP0304, TP0433 (*arp*), TP0470 and TP0967, and IGR TP0488-9 were done using R (R Core Team [40]; Vienna, Austria) (packages DescTools [41] and dplyr [42]). Scatter plots were constructed using Prism5 (GraphPad, San Diego, USA), and the number of repeats in these genes was checked for normality using the Shapiro-Wilk test (if p-values were > 0.05, we assumed normality), and p-values were determined using the Unpaired Two-sample t-test for data with a normal distribution or Mann-Whitney test for data without a normal distribution (difference was considered statistically significant if the p-value was < 0.05).

Phylogenetic trees were constructed using MEGAX (v.10.2.6, [43]) with 1000 replicates. Genes identified as those with positively selected sequences [44] were removed from the whole genome sequence alignments, as well as all *tpr* genes, TP0433 (*arp)*, TP0470, and both t0012 and t0015 (*tRNA-Ala* and *tRNA-Ile*), due to the previously published presence of recombination and/or positive selections. We inferred the evolutionary history through the application of the Maximum Likelihood method using the Tamura-Nei model [45]. Initial tree(s) for the heuristic search were obtained automatically by applying Neighbor-Joining (NJ) and BioNJ algorithms to a matrix of pairwise distances estimated using the Tamura-Nei model and then selecting the topology with superior log-likelihood values. All positions containing gaps and missing data were consequently removed from the analysis. We also constructed a phylogenetic tree using draft genome sequences of strains 11LMF5200815 and 49F8190407, as well as from other published *TPE* genomes of NHP from Cote d’Ivoire [13], Cote d’Ivoire, Gambia, Senegal, and Tanzania [11] and from Senegal [12], and from *TPE* of human origin, i.e., from Lihir Island [46], Solomon Islands [47], and Liberia [48]. Genome sequences were produced from available SRA data using the same approach as described above using Bioinformatic analysis, with the exception of genomes originating from study of Mediannikov (2020) [12], where only draft assemblies are publicly available. From the available 68 draft genome sequences, 59 had a broad genome length coverage (of more than 3 reads) of 92.8% or higher. Sequences with less coverage were not used for the construction of phylogenetic trees.

Pseudogenes were analyzed in all *TPE* genes with full genome sequences (n = 18). The following criteria were applied: (1) predicted gene length longer than 415 bp to avoid misannotated open reading frames, (2) nucleotide variants in the first 100 bps leading to frameshift or premature stop codons were not considered due to the presence of a possible downstream start of the gene, and (3) nucleotide variants in the last quarter of the gene were not considered due to the possibility of a variable C-terminus of the mature protein.

Whole genome sequences were annotated using Geneious Prime v2021.1.1 (Biomatters Ltd., Auckland, New Zealand).

## Results

### Characteristics of *TPE* isolates and whole-genome sequencing parameters

All *NHP* TPE strains analyzed in this study are shown in Table 3. Seven samples originated from *Papio anubis*, and three samples were collected from *Chlorocebus pygerythrus*. Of these, we were able to generate complete genome sequences from five *P*. *anubis* and three *C*. *pygerythrus* infecting strains.

**Table 3:** *Treponema pallidum* subspecies *pertenue* (*TPE*) strains analyzed in this study.

*TPE* isolate	Source	Sex	Origin	Year of isolation	gDNA concentration (ng/μl)	No. of *TPE* copies per μl	DNA enrichment method	No. of mapped reads	Genome coverage (%)	Average coverage	Complete finished genome sequence (% of genome covered)
6RUM2090716	*C*. *pygerythrus*	M	RNP	2016	1682	nd	Looxter^*a*^	17,006,986	98.65	2274x	Yes (100%)
7SNM5081115	*P*. *anubis*	M	SNP	2015	1659	nd	Looxter	978,074	98.47	131x	Yes (100%)
11LMF5200815	*P*. *anubis*	F	LMNP	2015	564	5.41E+03	*Dpn*I^*b*^, PSGS^*c*^	24224732	92.90	3185x	No (93%)
18NCF8220317	*P*. *anubis*	F	NCA	2017	209	6.50E+03	*Dpn*I	1,340,910	98.58	157x	Yes (100%)
19LMF8280815	*P*. *anubis*	F	LMNP	2015	1002	4.17E+04	*Dpn*I	185,342	98.08	19x	Yes (100%)
22LMF5290815	*P*. *anubis*	F	LMNP	2015	1652	nd	Looxter	554,102	98.54	122x	Yes (100%)
24SNM5151115	*P*. *anubis*	M	SNP	2015	1586	nd	Looxter	1,008,626	98.34	135x	Yes (100%)
32LMM2190317	*C*. *pygerythrus*	M	LMNP	2017	233	1.47E+05	*Dpn*I	3,756,864	98.58	435x	Yes (100%)
34LMM2190317	*C*. *pygerythrus*	M	LMNP	2017	164	6.39E+05	*Dpn*I	2,711,410	98.80	325x	Yes (100%)
49F8190407	*P*. *anubis*	F	LMNP	2007	1681	nd	Looxter	225412	98.19	30x	No (98%)

nd, not determined; M–male, F–female; SNP—Serengeti National Park, TZ; NCA—Ngorongoro Conservation Area, TZ; LMNP—Lake Manyara National Park, TZ; RNP—Ruaha National Park, TZ; GSNP—Gombe Stream National Park, TZ

^*a*^ DNA enriched by LOOXTER Enrichment Kit (Analytik Jena, Germany)

^*b*^ performed according to Grillová *et al*., [18]

^*c*^ PSGS performed according to Weinstock *et al*., [20], an overview of obtained sequences is shown in S1 Table.

All raw sequence read files can be found in NCBI under BioProject numbers PRJNA813752, PRJNA813753, PRJNA813756, PRJNA813757, PRJNA813758, PRJNA813760, PRJNA813762, PRJNA813764, PRJNA820556 and PRJNA820558. GenBank accession numbers for the eight complete annotated sequences can be found in S1 Data–Comparison of genomic features.

### Comparison of *TPE* genomes of human and NHP origin

The genomes of *TPE* isolates infecting NHPs determined in this study were compared to available whole genome sequences of *TPE* of human origin. Eight human *TPE* genomes with complete genome sequences were available: strains Samoa D (CP002374), Gauthier (CP002376), and CDC-2 (CP002375) [49], strains CDC-2575 (CP020366) and Ghana-051 (CP020365) [50], strains Kampung Dalan K363 (CP024088) and Sei Geringging K403 (CP024089) [51], as well as strain CDC-1 (CP024750, this study). In addition, two previously completed whole-genome sequences originating from NHP infecting strains (strain Fribourg-Blanc, CP003902 [10] and strain LMNP-1, CP021113 [11]) were included.

**Fig 2:**
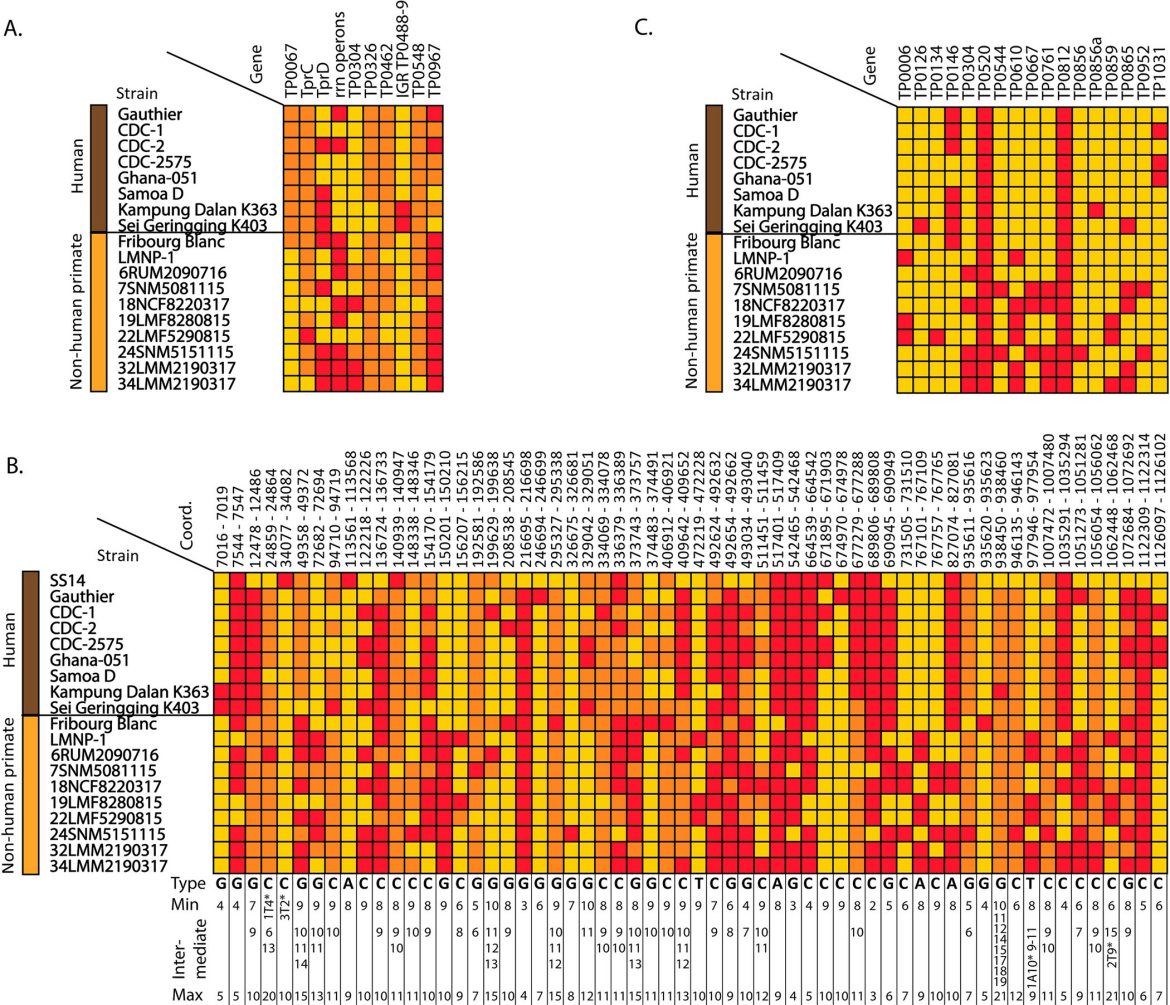
Comparison of human and NHP strains. Samples of human and NHP origin are marked in brown and yellow, respectively. (A) A comparison of genomic features between *TPE* of human and nonhuman primate origin. Only genomic regions showing similar differences and detected in two or more genomes are shown. For more details see S1 Data–Comparison of genomic features. Same color cells indicate the same sequence variant. For changes present only in one genome see S2 Table. (B) Differences in the length of homopolymeric tracts in the analyzed genomes. Coordinates correspond to the reference genome LMNP-1. The length of homopolymeric tracts was determined by the dominating variant in the sequencing reads of any given homopolymer. Homopolymeric tracts differing in the number of nucleotides in at least one genome are shown. For comparison, the length of homopolymeric tracts in the SS14 genome is shown. The maximum (red) and minimum (yellow) number of nucleotides are shown, with all values in between shown in orange. All length values are listed at the bottom of the figure. Exact numbers of nucleotides in homopolymeric tracts for each genome that have three or more variants are shown in S1 Fig. *Homopolymers with nucleotide substitution. 1T4 = CTC CCC, 3T2 = CCC TCC, 1A10 = CAC CCC CCC CCC, 2T9 = CCT CCC CCC CCC. (C) Pseudogenes identified in the set of analyzed *TPE* strains/isolates. Identified pseudogenes are highlighted in red while yellow indicates functional genes.

The comparison of genomic features, including indels, pseudogenes, differences in gene alleles between humans and NHP *TPE* (S1 Data–Comparison of genomic features), and differences in the length of homopolymeric tracts are shown in Fig 2. Altogether, 13 genomic regions in *TPE* genomes of either NHP or human origin showed variants that were detected in two or more genomes (S1 Data–Comparison of genomic features). These differences included (1) different alleles of the *tpr*C and *tpr*D genes, (2) variants of *rrn* operons, (3) indels in TP0067, TP0136, TP0326, TP0462, and TP0548, and (4) the number of repeats in TP0304, TP0433, TP0470, IGR TP0488-489, and TP0967. Genetic differences found only in a single genome are shown in S2 Table.

**Fig 3:**
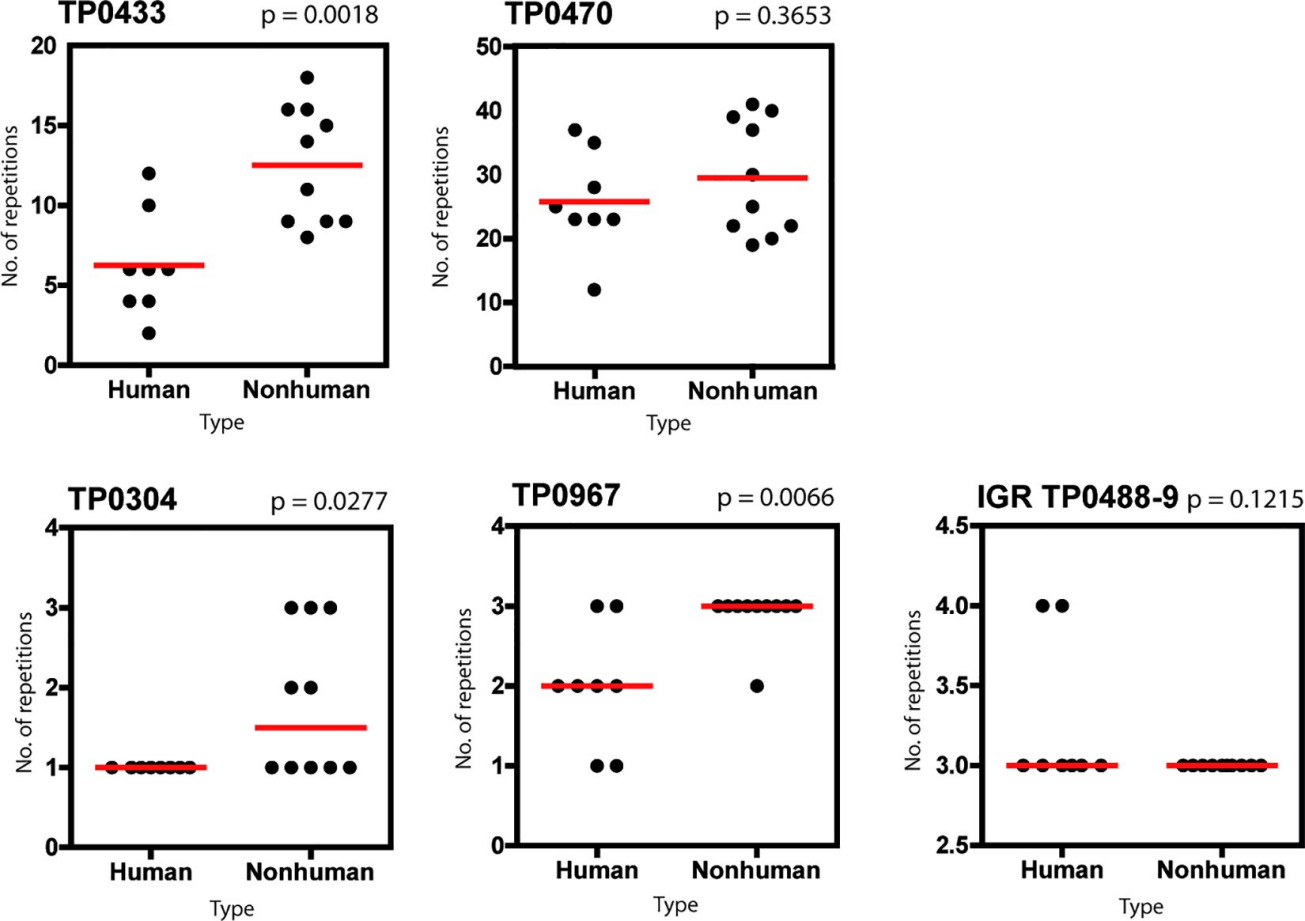
The number of repeats in genes TP0304, TP0433 (*arp*), TP0470, TP0967, and IGR TP0488-9 showed differences between *TPE* strains of human and NHP origin. The mean values (marked by red lines) for TP0433 and TP0470 genes with normal data distribution were 6.25 and 12.5 and 25.8 and 29.5 for *TPE* isolates from humans and NHPs, respectively. The median values (marked by red lines) for genes TP0304, TP0967, and IGR TP0488-9, which did not have normal data distribution, were 1 and 1.5, 2 and 3 and 3 and 3, for *TPE* isolates from humans and NHPs, respectively. The differences in the TP0470 and IGR TP0488-9 genes were not statistically significant (p-value > 0.05). The p-values are shown at the top of column scatter plots for individual TP0304, TP0433, TP0470, TP0967, and IGR TP0488-9 genes.

No genomic differences reflecting the origin of *TPE* strains (either NHP or human) were found between the analyzed genomes. However, most of the *TPE* genomes of NHP origin, except for the Fribourg-Blanc genome, contained a 9 nt-long deletion in the repeat-containing region of the TP0067 gene. The mean (or median) number of repeats in genes TP0304, TP0433 (*arp*), TP0470, and TP0967 were higher in *TPE* strains that infect NHPs, and this difference was statistically significant (p-value < 0.05) for the TP0304, TP0433, and TP0967 genes (Fig 3). The number of *arp* repeats in *TPE* genomes from NHPs had higher average lengths (14.3 repeats), while the human *TPE* strains analyzed to date contained, on average, 6.25 repeats per *arp* gene (S1 Data–Comparison of genomic features). The difference in the number of repeats in TP0470 and intergenic region (IGR) TP0488-489 was not statistically significant between *TPE* isolates from humans and NHPs.

In addition to the number of repeats, differences in the length of homopolymeric tracts were also analyzed (Fig 2B). There were no differences between *TPE* strains that infect humans or those that infect NHPs, with the single exception of a difference in the homopolymeric tract within the TP0012 gene between coordinates 12,478–12,486. In this homopolymer, all *TPE* strains from NHPs differed from human *TPE* in their homopolymeric tract lengths (Fig 2B).

Among the set of analyzed *TPE* strains/isolates with finished genome sequences, 17 pseudogenes were identified (Fig 2C) based on criteria specified in the Material and Methods section. Pseudogenes in TP0520 and TP0812 were found in all genomes. Although the analyzed genomes of *TPE* from NHP origin showed more frequent occurrences of pseudogenes, no consistent differences in the presence of pseudogenes were found between *TPE* genomes from NHPs and humans. The Fribourg-Blanc isolate showed the presence of the same number of pseudogenes as that found in *TPE* strains of human origin.

### Analyses of *tpr* genes

**Fig 4:**
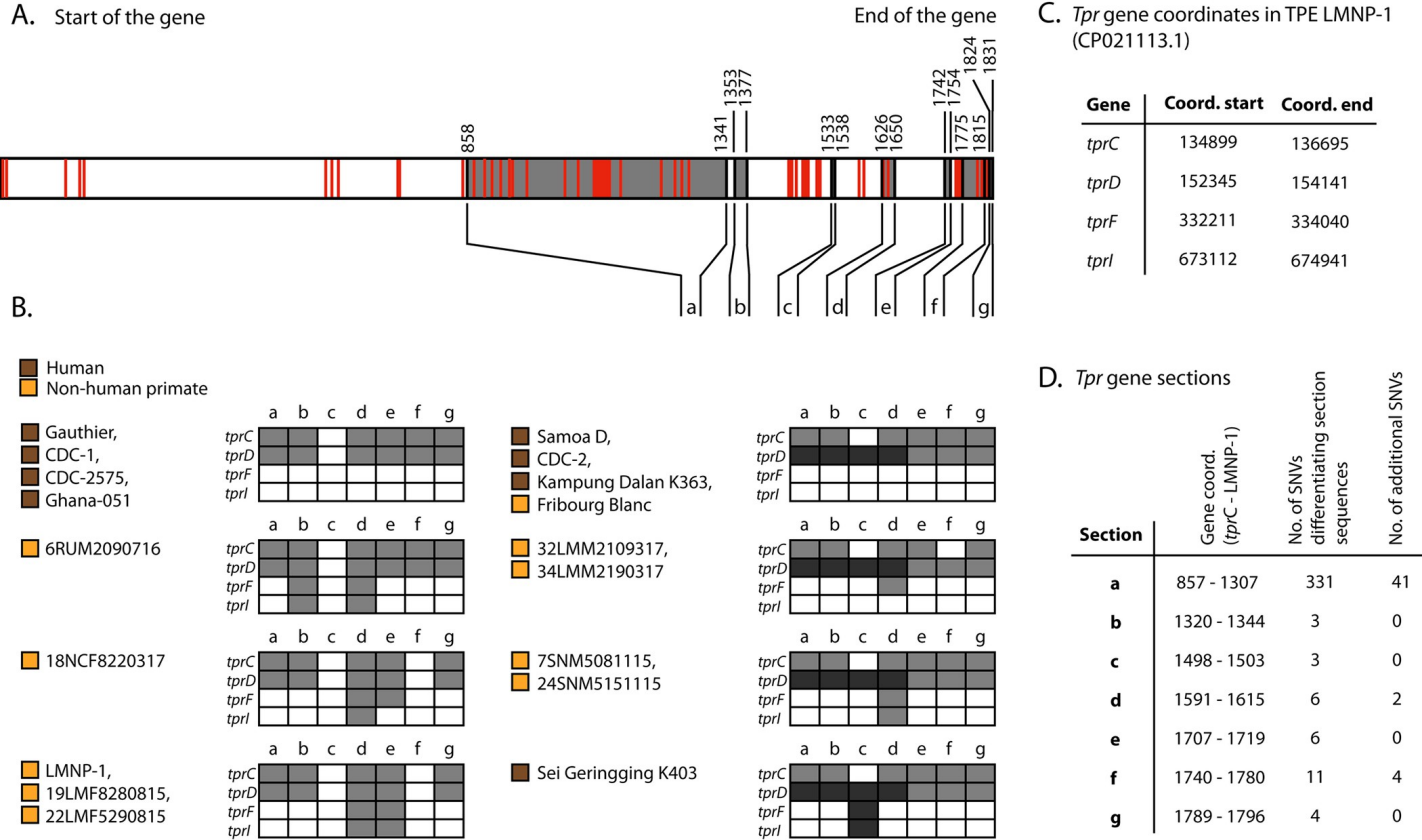
Sequence analysis of *tpr*C, D, F, and I genes. **A.** Visualization of the full-length *tpr*C, D, F, and I genes, including gene sequences marked as sections (a–g, shown in grey, seven in total). Each section was defined as a DNA region containing two or more nucleotide sequences that differ in three and more positions. Black lines denote positions of single nucleotide variants (SNVs) at the start and at the end of each section. Single nucleotide variants not following the pattern of the section detected within and outside sections are shown as red lines. **B.** Modular structure of *tpr*C, D, F, and I genes. White and grey show the two different versions of the DNA regions. Dark grey represents the tprD2 allele. Different sequence versions of the sections are combined into different patterns of the *tpr*C, D, F, and I genes in different *TPE* isolates. In general, the *tprF* and *tprI* genes in *TPE* isolated from NHPs show more variability in combining different versions of the sections compared to *TPE* of human origin. The Fribourg-Blanc isolate, CDC-2, Samoa D, and Kampung Dalan K363 have identical modular structures of *tpr*C, D, F, and I. **C.** Coordinates of the *tpr*C, D, F, and I genes with respect to the LMNP-1 reference. **D.** Section coordinates according to *tprC* gene coordinates of LMNP-1 reference and the number of SNVs differentiating sections as well as the number of additional SNVs.

The number of variable nucleotide sites ranged between two to five in individual *tpr*A, B, E, G, H, J, and L genes. The *tpr*K gene contained intrastrain heterogeneity sites [52] and was not analyzed in this study. The remaining *tpr*C, D, F, and I genes had considerably higher variability compared to *tpr*A, B, E, G, H, J, and L genes in *TPE* genomes of both human and NHP origin. The *tpr*D gene showed the presence of two alleles (*tpr*D and *tpr*D2; S1 Data–Comparison of genomic features) with several single nucleotide variants (SNVs) found predominantly within the *tpr*D allele (S2 Fig). The *tpr*C, D, F, and I genes were found to contain a modular structure with seven identifiable sections (a–g) (Fig 4), as previously suggested by Strouhal *et al*. [51]. Each section was defined based on nucleotide sequences differing in three and more positions. The number of nucleotide differences was 331 for section a, 3 for section b, 3 for section c, 6 for section d, 6 for section e, 11 for section f, and 4 for section g. The number of SNVs present inside each section (irrespective of section pattern) was 41, 0, 0, 2, 0, 4, and 0, in sections a-g, respectively. Another 74 SNVs were located outside of the sections a–g. Each section was present in two variants, except for sections a, b, and d, which were present in three variants. Different sequence variants of the sections combined into different patterns (n = 8) of the *tpr*C, D, F, and I genes in different TPE isolates. Generally, *tpr*F and *tpr*I genes in TPE isolated from NHPs showed more variability (Fig 4) and combined more different *tpr* sections. Altogether, more patterns, including all *tpr*C, D, F, and I genes, were detected in NHPs (n = 6) compared to patterns in TPE of human origin (n = 3). One combination pattern was present in both groups. No consistent patterns were observed to differentiate between the two groups. The Fribourg-Blanc isolate resembles human *TPE* strains (CDC-2, Samoa D, Kampung Dalan K363).

### Phylogenomic analysis

**Fig 5:**
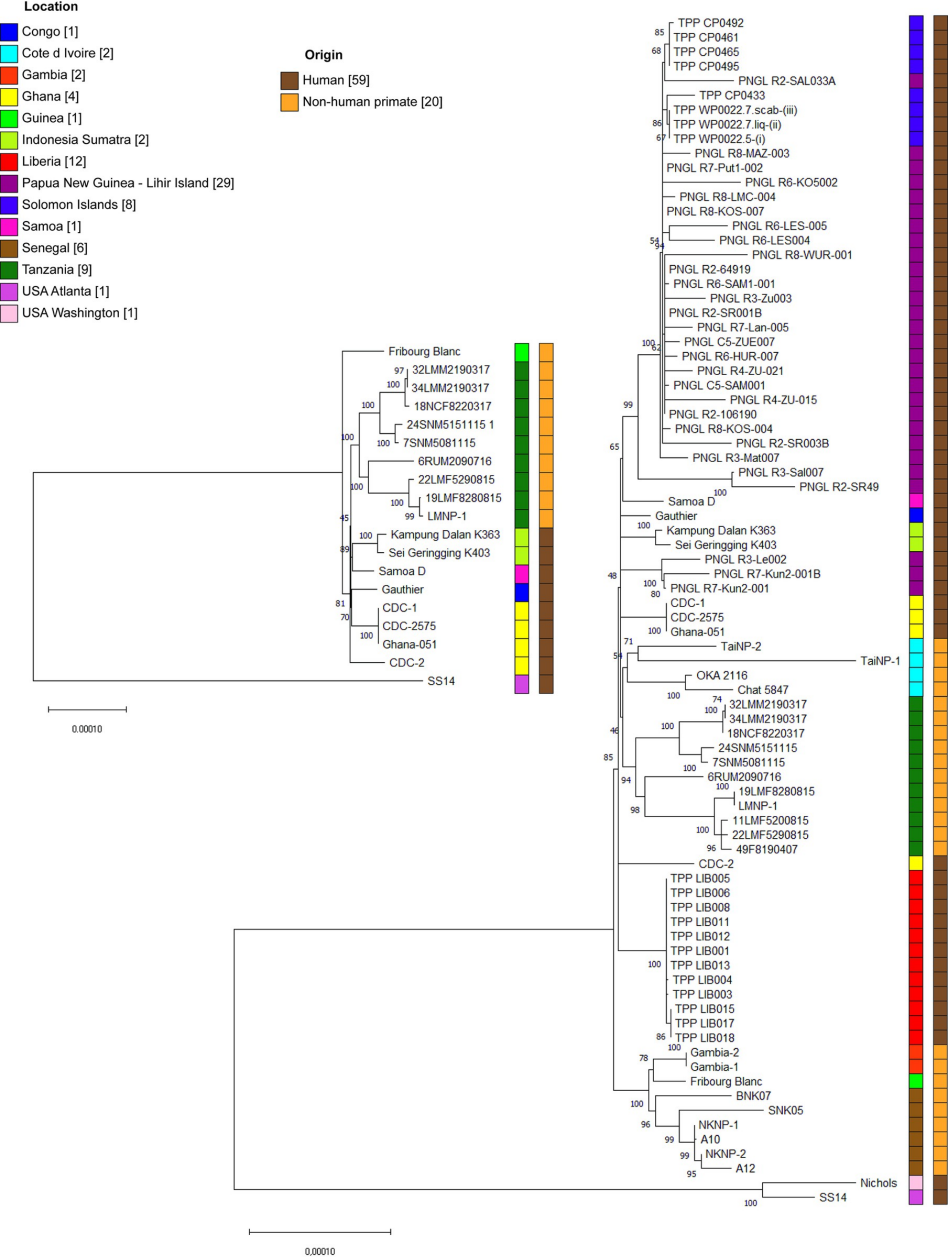
Phylogenetic analysis of *TPE* genomes of human and NHP origin. The phylogenetic relatedness of *TPE* genomes determined for complete (left tree) and draft (right tree) genomes. Draft genomes from other studies were used [11–13,46–48]. Draft genomes with more than 83,7% coverage were used for tree construction. *TPA* strains SS14 and Nichols were used as outgroups. For more details see S1 Data–Overview of strains (phylogeny). The evolutionary history was inferred by using the Maximum Likelihood method and Tamura-Nei model [45]. The bootstrap values of trees in which the associated taxa clustered together is shown next to the branches. The tree is drawn to scale, with branch lengths measured in the number of substitutions per site. All positions containing gaps and missing data were deleted. There was a total of 1,092,985 and 651,557 positions in the tree containing 19 and 80 nucleotide sequences, respectively, with 1561 and 4311 variable sites. The color legend next to the trees marks the geographical origin of the samples. Human and nonhuman primate origin is displayed using brown and yellow color, respectively. *TPE* genomes predominantly cluster relative to the geographical origin.

To assess the phylogenetic relatedness of the analyzed *TPE* genomes, recombinant and positively selected regions identified in a previous study on *TPE* genomes [44] were removed, as well as all *tpr* genes, TP0433 (*arp)*, TP0470, and both t0012 and t0015 (*tRNA-Ala* and *tRNA-Ile*), and the remaining sequences were aligned and used for construction of a phylogenomic tree (Fig 5). *TPE* strains of human and NHP origin cluster into distinct groups (although with low bootstrap support for the human clade), while the Fribourg-Blanc strain falls as a sister lineage to all other *TPE* strains. Although this tree shows separation by host origin, it contains a limited subset of genomes and all but one NHP strain originate from Tanzania. We extended our analysis by including *TPE* draft genome sequences from other studies to better generate a broader phylogenetic picture. The draft genome sequence-based tree shows a clustering based on geographical origin and not based on host species. In this tree, the Fribourg-Blanc strain clustered with other samples from West Africa. This finding is in agreement with previous studies [11,13,46,48].

## Discussion

Despite the fact that our study is limited in extent, it contributes additional eight complete and two draft genome sequences of NHP infecting *TPE* isolates from Tanzania to the current set of available *TPE* genomes. The selected samples were not randomly chosen but instead represented genetically divergent samples isolated from different NHP species and geographical areas (Fig 1) [15]. Since isolates from the same geographical region often show genetic relatedness (Fig 5), preselection of samples based on multi-locus sequence typing allowed us to overcome the risk of sequencing clonal isolates [46–48,53].

In this study, the intrastrain heterogeneity analyzing variability within the infecting treponemal population was not determined. Instead, we used the most prevalent nucleotide variants at any given position.

We showed that *TPE* genomes isolated from NHPs do not show consistent genomic differences from *TPE* strains that infect humans. This indicates that current NHP *TPE* strains are not only similar [11] but belong to the same subspecies as human *TPE*. Previous studies have supported this finding based on a single complete genome [10,11] or draft genomes [11,12] as well as MLST [15]. In our study, we present several whole genome *TPE* sequences of NHPs that do not show consistent differences compared to human *TPE* strains. This finding indicates that these strains are indeed the same subspecies. It includes the largest dataset of primate (human and nonhuman) closed *TPE* genomes to date–eight *TPE* genomes of human origin [21,50,51] (CP024750) and ten *TPE* whole-genome sequences of NHP origin from Africa [10,11]. The number of investigated genomes is still limited, plus all samples originate from Tanzania. However, we are confident that this does not affect our main statement, which is that NHP infecting strains are not genetically distant from human-infecting TPE strains. All genomes included into the study have the same genetic characteristics.

We were able to resolve previously undetermined *TPE* chromosomal regions (sequencing gaps) that prevented us from making a conclusion regarding the sequence identity of *TPE* genomes from NHPs and humans since we were not able to rule out the potential existence of substantial differences in the previously unknown parts of the genome. While strains with different characteristics can exist in unsampled datasets, identification of strains that do not differ from strains infecting humans proves that NHP populations can serve as a potential reservoir for such infection at least in the studied area. This is further supported by the fact that, while highly unethical, experimental inoculation of the Fribourg-Blanc strain into human skin resulted in a viable yaws infection [54]. Likewise, experimental inoculation of NHPs with *TPE* of human origin resulted in typical yaws skin lesions [55].

Our current study seems to complicate yaws eradication efforts, in particular, because of the high *TPE* infection rates among African NHPs [11,12,15,56] where in some of them, clinical and genomic evidence of yaws-like lesions has been demonstrated [14]. However, the risk of a zoonotic spillover is difficult to proof. It is still unclear whether human and nonhuman primate infecting strains are epidemiologically linked. If, for example, NHPs frequently functioned as a source for human infection, we would see infections in children in Tanzania, which we demonstrated is not the case [57]. The reason that *TPE* strains from NHPs cluster separately from current human *TPE* of human origin (Fig 5) is also supported by previous studies [11,12,15] and is likely the effect of the different geographic sampling locations; however, it could also indicate infrequent spillovers between humans and NHPs. Similar genome characteristics (S1 Data–Comparison of genomic features, Fig 4) between the Fribourg-Blanc (of NHP origin) and human CDC-2 strains suggest that such transfers have happened in the past. For NHP *TPE* strains, the interspecies transmission of *TPE* has already been suggested [15]. So far, sample sizes from any geographic region are insufficient, and future studies are warranted to collect more *TPE* samples from areas where infected humans and NHPs coexist.

As predicted earlier [52] and shown in this study, the *tpr*C, D, F, and I genes showed a modular structure and were found to be more genetically diverse in *TPE* of NHP origin than the human *TPE* strains. The 3’ gene half encodes for a C-terminal domain MOSP^C^ having a β-barrel structure residing in the outer membrane [58]. A possible explanation for the observed higher variability among *TPE* in NHPs could be that (wild) NHPs are generally not treated for infection, which favors natural selection of new variants of outer membrane proteins while human infections are more often treated with antibiotics.

Significantly higher numbers of repeats in the *arp* gene (TP0433) among the NHP isolates were found compared to human *TPE* strains. We showed that NHP *TPE* has an average length of 14.3 *arp* repeats, which is similar to the most frequent number of repeats in human *TPA* strains [59]. Moreover, while there is an inversed correlation between the number of detected repeats in the *arp* and TP0470 genes, as shown by linear regression analysis among human *TPE* strains [60], no such correlation was observed for NHP *TPE*; interestingly, this is also seen in human *TPA* (syphilis). While the reason for these observations is unclear and could potentially reflect sexual transmission [61], the difference in the number of *arp* repeats, and any other repeat regions (S1 Data–Comparison of genomic features) found in the genome, should not be considered as the main difference between human and NHP *TPE* genomes. This is because the extension and shortening of sequences containing repetitive motifs are one of the most frequent changes observed in pathogenic treponemes and other bacteria [62]. We should also not forget that the number of repeats is highly mutable, as seen in human syphilis cases with two parallel samples collected at the same time (skin swab and whole blood sample) that showed two different *arp* repeat numbers [63]. The number of *arp* repeats, therefore, represents a variable and fast adaptation to a particular host, transmission route, and/or climate conditions.

## Conclusion

Our detailed analysis of *TPE* genomes revealed no consistent genomic differences between *TPE* genomes from human and NHP infections, thus demonstrating that NHP *TPE* isolates are not genomically distinct from strains causing human yaws. Although interspecies transmission seems to be rare and evidence for current spillover events are missing, the existence of *TPE* in NHPs is clearly demonstrated. There is no doubt that the endemic character of human yaws infection together with the presence of effective single peroral azithromycin therapy [4,64,65] promised successful and straightforward yaws elimination from the human population [7]. Full eradication of *TPE* would, however, require treatment of African NHPs, which cannot be realistically achieved. The low risk of spillover supports the current yaws treatment campaign, which under no circumstances should be stopped. Instead, yaws eradication in humans must be followed by continuous disease surveillance in areas where NHP infection is known to be present.

## Supporting information

S1 Table The results of PSGS sequencing of the 11LMF5200815 genome. (XLSX)

S2 Table Changes specific for a single genome out of the 18 analyzed genomes. (XLSX)

S1 Fig Homopolymers found among analyzed strains with three or more variants in homopolymeric tract length. (TIF)

S2 Fig Variable positions identified in the *tpr*D gene. The major difference in variable positions differentiated *tpr*D and *tpr*D2 alleles. Only variable positions are shown. Deletions are shown in white, variants in dark grey, and difference in the nucleotide variant is shown in black.
(TIF)

S1 Data List of gaps + primers. List of sequencing gaps and primers used for gap filling. **Comparison of genomic features.** A comparison of genomic features between *TPE* of human and nonhuman primate origin. Only genomic regions showing similar differences from other genomes and detected in two or more genomes are shown. Indels and sequence differences found only in single genomes are not displayed. Shaded cells indicate the same sequence variant. **Overview of strains (phylogeny).** Details of the strains used for phylogenetic analysis.
(XLSX)
