# Supplementary figures and images for: The genomes of the yaws bacterium, Treponema pallidum subsp. pertenue, of nonhuman primate and human origin are not genomically distinct
Source: PLoS Negl Trop Dis. 2023 Sep 13;17(9):e0011602. doi: 10.1371/journal.pntd.0011602 (PMC10499264; doi:10.1371/journal.pntd.0011602)

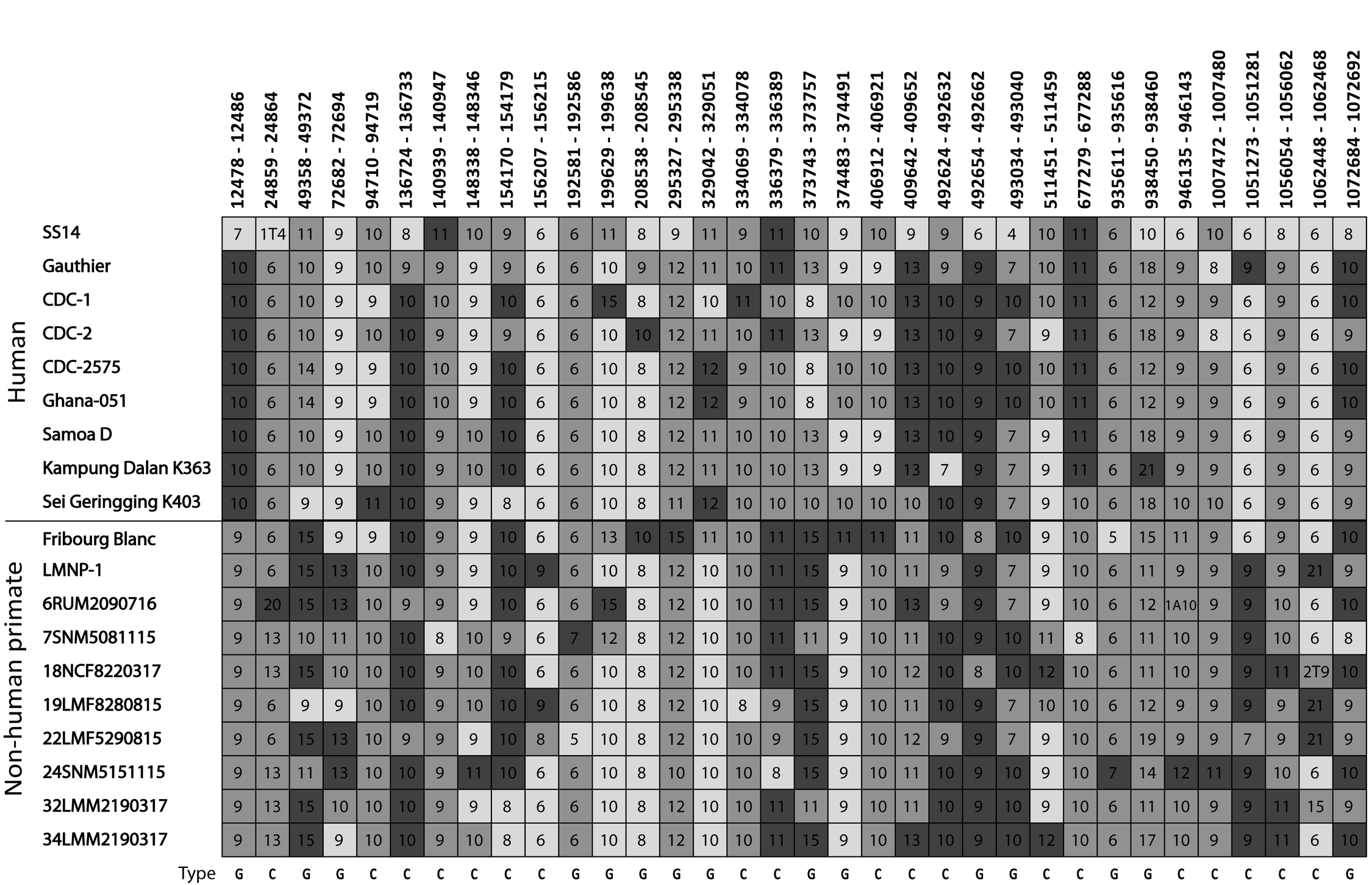

Supplement: S1 Fig — (TIF) [file pntd.0011602.s003.tif]

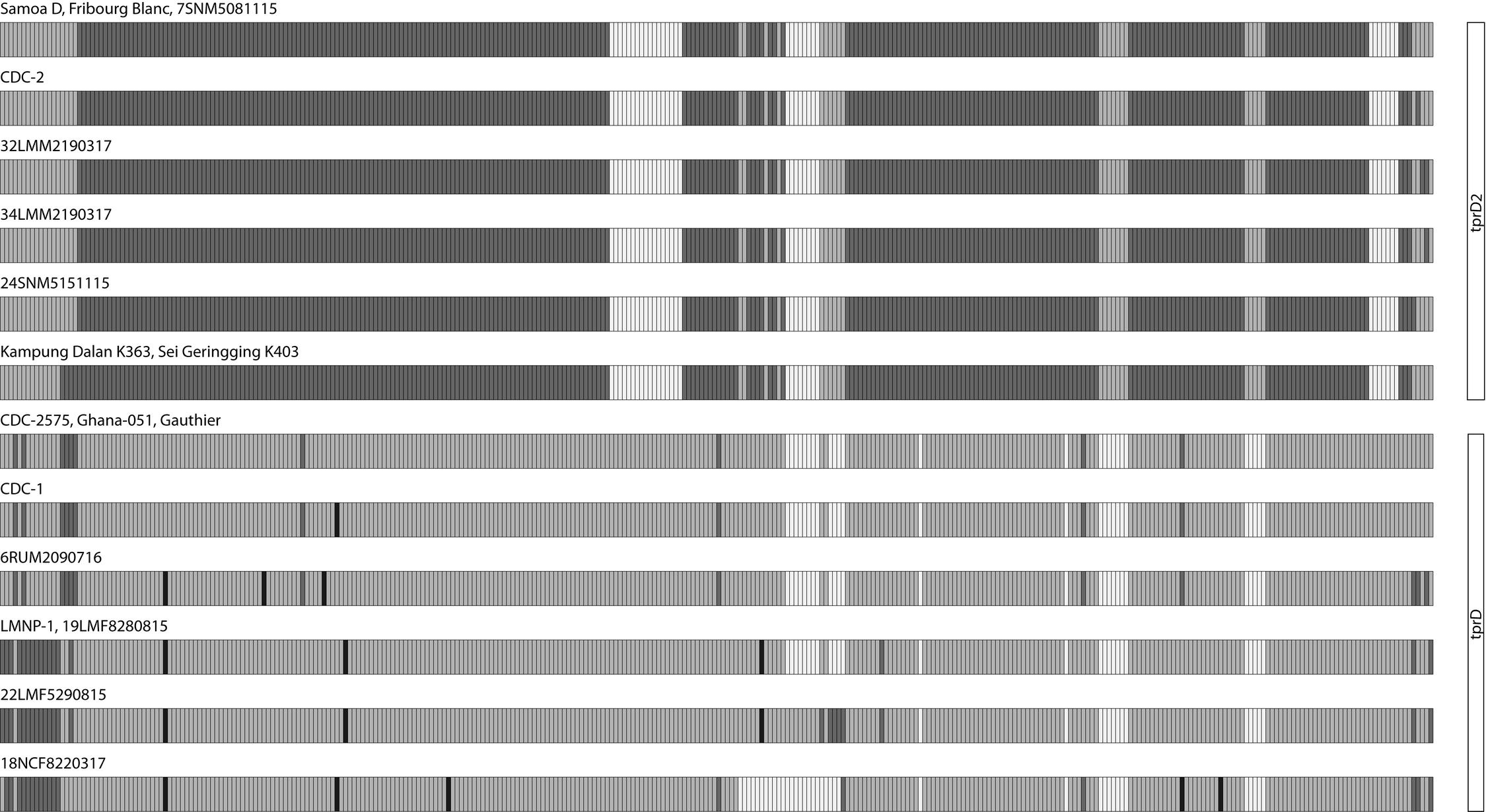

Supplement: S2 Fig — The major difference in variable positions differentiated tprD and tprD2 alleles. Only variable positions are shown. Deletions are shown in white, variants in dark grey, and difference in the nucleotide variant is shown in black. (TIF) [file pntd.0011602.s004.tif]
